# Supplementary material for: Depletion of Hemoglobin Transcripts and Long-Read Sequencing Improves the Transcriptome Annotation of the Polar Bear (Ursus maritimus)
Source: Front Genet. 2019 Jul 19;10:643. doi: 10.3389/fgene.2019.00643 (PMC6658610; doi:10.3389/fgene.2019.00643)
Supplement: Supplementary file 1 [file DataSheet_1.pdf]

Supplementary Data

for

Depletion of hemoglobin transcripts and long read sequencing improves the transcriptome annotation of the polar bear (*Ursus maritimus*)

Ashley Byrne[1,2], Megan A. Supple[3], Roger Volden[2,4],Kristin Laidre[5], Beth Shapiro[3,6], Christopher Vollmers[2,4]

Includes:

Figues S1-S4
Tables S1 and S2

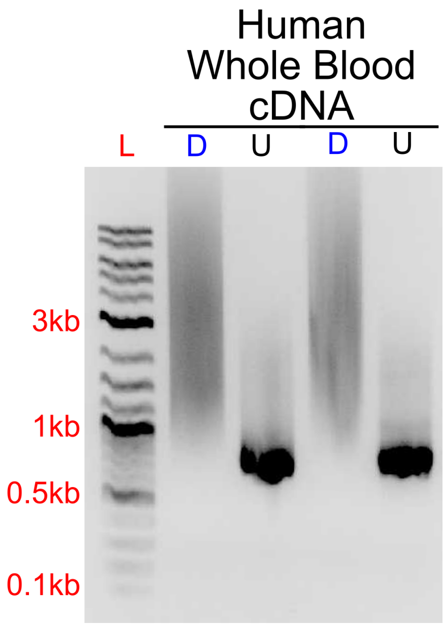

Figure S1: ***Long-DASH sgRNA also depletes human hemoglobin transcripts from full-lengthcDNA.*** *Technical replicates of depleted (D) or undepleted (U) human whole blood cDNA were visualized on an agarose gel. DNA ladder (L) suggests highly abundant cDNA species - putatively hemoglobin around ~700bp.*

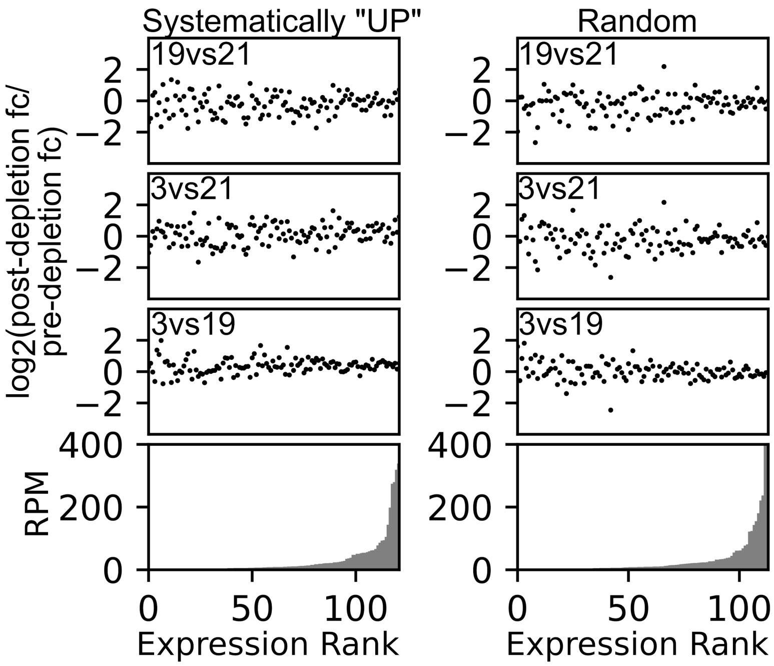

Figure S2: ***Depletion of hemoglobin affects expression levels within but not fold-change between samples***
Expression levels(bottom) as well as the changes in differential expression between polar bears pre- and post-depletion are shown for genes that are upregulated in all 3 polar bears post-depletion (Systematically "UP") and a random selection of genes with similar expression distribution (Random).

A) Hemoglobin, alpha (HBA)

```
XM_008696690.1          --------------------CCGCCCCGCACATTTCTGGTCCTCACAGACTCAGAAAGAA    40
NM_000558.4             CATAAACCCTGGCGCGCTCGCGGCCCGGCACTCTTCTGGTCCCCACAGACTCAGAGAGAA    60
NM_000517.4             CATAAACCCTGGCGCGCTCGCGGGCCGGCACTCTTCTGGTCCCCACAGACTCAGAGAGAA    60
                                            * * ** ****  ********* ************ ****

XM_008696690.1          GCCACCATGGTGCTGTCTCCCGCCGACAAGAGCAACGTCAAGGCCACCTGGGATAAGATC    100
NM_000558.4             CCCACCATGGTGCTGTCTCCTGCCGACAAGACCAACGTCAAGGCCGCCTGGGGTAAGGTC    120
NM_000517.4             CCCACCATGGTGCTGTCTCCTGCCGACAAGACCAACGTCAAGGCCGCCTGGGGTAAGGTC    120
                         ******************* ********** ************* ****** **** **

XM_008696690.1          GGCAGCCACGCTGGCGAGTATGGCGGCGAGGCTCTGGAGAGGACCTTCGCGTCCTTCCCC    160
NM_000558.4             GGCGCGCACGCTGGCGAGTATGGTGCGGAGGCCCTGGAGAGGATGTTCCTGTCCTTCCCC    180
NM_000517.4             GGCGCGCACGCTGGCGAGTATGGTGCGGAGGCCCTGGAGAGGATGTTCCTGTCCTTCCCC    180
                        ***   ***************** *  ***** **********  ***  **********

XM_008696690.1          ACCACCAAGACCTACTTCCCCCACTTCGACCTGAGCCCTGGCTCCGCCCAGGTCAAGGCC    220
NM_000558.4             ACCACCAAGACCTACTTCCCGCACTTCGACCTGAGCCACGGCTCTGCCCAGGTTAAGGGC    240
NM_000517.4             ACCACCAAGACCTACTTCCCGCACTTCGACCTGAGCCACGGCTCTGCCCAGGTTAAGGGC    240
                        ******************** ****************  ***** ******** **** *

XM_008696690.1          CACGGCAAGAAGGTGGCCGACGCCCTGACCACCGCCGCAGGCCACCTGGACGACCTGCCG    280
NM_000558.4             CACGGCAAGAAGGTGGCCGACGCGCTGACCAACGCCGTGGCGCACGTGGACGACATGCCC    300
NM_000517.4             CACGGCAAGAAGGTGGCCGACGCGCTGACCAACGCCGTGGCGCACGTGGACGACATGCCC    300
                        *********************** ******* *****  *  *** ******** ****

XM_008696690.1          GGCGCCCTGTCCGCTCTGAGCGACCTGCACGCGCACAAGCTGCGAGTGGACCCGGTCAAC    340
NM_000558.4             AACGCGCTGTCCGCCCTGAGCGACCTGCACGCGCACAAGCTTCGGGTGGACCCGGTCAAC    360
NM_000517.4             AACGCGCTGTCCGCCCTGAGCGACCTGCACGCGCACAAGCTTCGGGTGGACCCGGTCAAC    360
                          *** ******** ************************** ** ***************

XM_008696690.1          TTCAAGTTCCTGAGCCACTGCCTGCTGGTGACCCTGGCCAGCCACCACCCCGCGGAGTTC    400
NM_000558.4             TTCAAGCTCCTAAGCCACTGCCTGCTGGTGACCCTGGCCGCCCACCTCCCCGCCGAGTTC    420
NM_000517.4             TTCAAGCTCCTAAGCCACTGCCTGCTGGTGACCCTGGCCGCCCACCTCCCCGCCGAGTTC    420
                        ****** **** ***************************  ***** ****** ******

XM_008696690.1          ACCCCTGCCGTCCACGCCTCCCTGGACAAGTTCTTCAGCGCCGTGAGCACCGTGCTCACC    460
NM_000558.4             ACCCCTGCGGTGCACGCCTCCCTGGACAAGTTCCTGGCTTCTGTGAGCACCGTGCTGACC    480
NM_000517.4             ACCCCTGCGGTGCACGCCTCCCTGGACAAGTTCCTGGCTTCTGTGAGCACCGTGCTGACC    480
                        ******** ** ********************* *  * *********************

XM_008696690.1          TCCAAATACCGTTAAGCTGGAGCCGCGCGACCCTCCCGCTCCCGGCCTGGGGCCTCTTGC    520
NM_000558.4             TCCAAATACCGTTAAGCTGGAGCCTCGGTGGCCATGCTTCTTGCCCCTTGGGCCTCCCCC    540
NM_000517.4             TCCAAATACCGTTAAGCTGGAGCCTCGGTAGCCGTTCCTCCTGCCCGCTGGGCCTCCCAA    540
                        ************************ **      ***  *         *   *******

XM_008696690.1          GC--------------TCCACGCGCCTGAACTTCCCGATCTTTGAATAAAGTCTGAGTGG    566
NM_000558.4             CAGCCCCTCCTCCCCTTCCTGCACCCGTACCCCCGTGGTCTTTGAATAAAGTCTGAGTGG    600
NM_000517.4             CGGGCCCTCCTCCCCTCCTTGCACC-GGCCCTTCCTGGTCTTTGAATAAAGTCTGAGTGG    599
                                         *      *     *  *  * **********************

XM_008696690.1          GCTGCAG--------------------    573
NM_000558.4             GCGGCAAAAAAAAAAAAAAAAAAAAAA    627
NM_000517.4             GCAGCAAAAAAAAAAAAAAAAAA----    622
                        **  **
```

B) Hemoglobin, beta (HBB)

```
XM_008709611.1          GAGCAGGGCCAGCTGCTGCTTATACTTGCTTCTGACACAACCGTGTTCACTAGCAACCAC    60
NM_000518.4             ---------------------ACATTTGCTTCTGACACAACTGTGTTCACTAGCAACCTC    39
                                             * * **************** **************** *

XM_008709611.1          AAAGAGACACCATGGTGCATCTGACTGGTGAGGAGAAGTCTCTCGTCACCGGCCTGTGGG    120
NM_000518.4             AAACAGACACCATGGTGCATCTGACTCCTGAGGAGAAGTCTGCCGTTACTGCCCTGTGGG    99
                        *** **********************  *************  *** ** * ********

XM_008709611.1          GCAAGGTGAACGTGGATGAAGTTGGTGGTGAGGCCCTGGGCAGGCTTCTGGTTGTCTACC    180
NM_000518.4             GCAAGGTGAACGTGGATGAAGTTGGTGGTGAGGCCCTGGGCAGGCTGCTGGTGGTCTACC    159
                        ********************************************** ***** *******

XM_008709611.1          CCTGGACTCAGAGGTTCTTTGACTCCTTTGGGGACCTGTCCTCTGCTGATGCTATTATGA    240
NM_000518.4             CTTGGACCCAGAGGTTCTTTGAGTCCTTTGGGGATCTGTCCACTCCTGATGCTGTTATGG    219
                        * ***** ************** *********** ****** ** ******** *****

XM_008709611.1          ACAACCCCAAGGTCAAGGCCCATGGCAAGAAGGTGCTGAACTCCTTTAGTGATGGCCTGA    300
NM_000518.4             GCAACCCTAAGGTGAAGGCTCATGGCAAGAAAGTGCTCGGTGCCTTTAGTGATGGCCTGG    279
                         ****** ***** ***** *********** *****     *****************

XM_008709611.1          AGAATCTGGACAACCTCAAGGGCACCTTTGCTAAGCTGAGCGAGCTGCACTGTGACAAGC    360
NM_000518.4             CTCACCTGGACAACCTCAAGGGCACCTTTGCCACACTGAGTGAGCTGCACTGTGACAAGC    339
                           * ************************** *  ***** *******************

XM_008709611.1          TGCACGTGGATCCCGAGAACTTCAAGCTCCTGGGCAACGTGCTGGTGTGTGTGCTGGCTC    420
NM_000518.4             TGCACGTGGATCCTGAGAACTTCAGGCTCCTGGGCAACGTGCTGGTCTGTGTGCTGGCCC    399
                        ************* ********** ********************* *********** *

XM_008709611.1          ACCACTTTGGCAAAGAGTTCACCCCTCAGGTGCAGGCTGCCTATCAGAAGGTGGTGGCTG    480
NM_000518.4             ATCACTTTGGCAAAGAATTCACCCCACCAGTGCAGGCTGCCTATCAGAAAGTGGTGGCTG    459
                        * ************** ******** *  ******************** **********

XM_008709611.1          GTGTGGCCAACGCCCTGGCCCACAAGTACCACTGAGCTCCTGGCCTGTTTCCTGGTGATC    540
NM_000518.4             GTGTGGCTAATGCCCTGGCCCACAAGTATCACTAAGCTCGCTTTCTTGCTGTCCAATTTC    519
                        ******* ** ***************** **** *****     **   *        **

XM_008709611.1          CCTG-GAAGACCCTGTTCCCCTAAATTCTATCTTCTGAACTGGGGGAAATAATGTCCACC    599
NM_000518.4             TATTAAAGGTTCCTTTGTTCCCTAAGTCCAACTACTAAACTGGGGGATATTATGAAGGGC    579
                          *   * *  *** *   **  ** ** * ** ** ********** ** ***     *

XM_008709611.1          ATCAAGGGTATGGTTTCTGCCTAATAAAGAACCTTCAGCTCAA----    642
NM_000518.4             CTTGAGCATCTGGATTCTGCCTAATAAAAAACATTTATTTTCATTGC    626
                         *  **  * *** ************** *** ** *  *  *
```

Figure S3. Alignment of orthologous HBA and HBB mRNA sequences in human and polar bear. *Multi-sequence alignment from Clustal Omega v1.2.4. * indicates a match. Underline and bold indicates target sequences used for sgRNA design for Globin depletion. Red indicates (N-GG) PAM sequence a) Hemoglobin, alpha (HBA) b) Hemoglobin, beta (HBB).*

A) Construction of sgRNAs

#1 tracrRNA oligo (in Reverse Orientation/Anti-sense)

```
   <-------------------tracrRNA-------------------------------- <-------Primer----->
5’-AAAAAGCACCGACTCGGTGCCACTTTTTCAAGTTGATAACGGACTAGCCTTATTTTAACTT GCTATTTCTAGCTCTAAAAC-3’
```

The oligo above is a universal tracrRNA template which allows you to generate full sgRNA templates with any target sequence oligo as long as the target sequence oligo meets the following requirements below:

1. The oligo contains the reverse complement of the primer sequence on the 3' end.
2. the oligo contains 'GG' on the 3' end of the target sequence for T7 RT.

#1 HBA1/HBA2 target oligos based upon mRNA prediction of Polar Bear/Human genes.

```
       <--------T7----------------><--target_sequence--><-------Primer------>
oligo 1 5'-GAAATTAATACGACTCACTATAGG AAGGSCCACGGCAAGAAGG GTTTTAGAGCTAGAAATAGC-3'
       <--------T7----------------><--target_sequence--><-------Primer------>
oligo 2 5'-GAAATTAATACGACTCACTATAGG CACTGCCTGCTGGTGACCC GTTTTAGAGCTAGAAATAGC-3'
       <--------T7----------------><--target_sequence--><-------Primer------>
oligo 3 5'-GAAATTAATACGACTCACTATAGG GGTYAAGGSCCACGGCAAGA GTTTTAGAGCTAGAAATAGC-3'
       <--------T7----------------><--target_sequence--><-------Primer------>
oligo 4 5'-GAAATTAATACGACTCACTATAGG ACCTCCAAATACCGTTAAGC GTTTTAGAGCTAGAAATAGC-3'
        <-------T7---------------><--target_sequence--><-------Primer------>
oligo 5 5'-GAAATTAATACGACTCACTATAGG GCCGACAAGASCAACGTCA GTTTTAGAGCTAGAAATAGC-3'
        <-------T7---------------><--target_sequence--><-------Primer------>
oligo 6 5'-GAAATTAATACGACTCACTATAGG GGGAAGTAGGTCTTGGTGGTG GTTTTAGAGCTAGAAATAGC-3'
        <-------T7---------------><--target_sequence--><-------Primer------>
oligo 7 5'-GAAATTAATACGACTCACTATAGG TCCTRAGCCACTGCCTGC GTTTTAGAGCTAGAAATAGC-3'
        <-------T7---------------><--target_sequence--><-------Primer------>
oligo 8 5'-GAAATTAATACGACTCACTATAGG CAGGTCGCTCAGRGCGGACA GTTTTAGAGCTAGAAATAGC-3'
```

#2 HBB target oligos based upon mRNA prediction of Polar Bear/Human gene.

```
        <-------T7----------------><--target_sequence--><-------Primer------>
oligo 1 5'-GAAATTAATACGACTCACTATAGG CACTGTGACAAGCTGCACG GTTTTAGAGCTAGAAATAGC-3'
       <-------T7----------------><--target_sequence--><-------Primer------>
oligo 2 5'-GAAATTAATACGACTCACTATAGG GAAGTTGGTGGTGAGGCCCT GTTTTAGAGCTAGAAATAGC-3'
       <-------T7----------------><--target_sequence--><-------Primer------>
oligo 3 5'-GAAATTAATACGACTCACTATAGG CAGGCTGCCTATCAGAARG GTTTTAGAGCTAGAAATAGC-3'
       <-------T7----------------><--target_sequence--><-------Primer------>
oligo 4 5'-GAAATTAATACGACTCACTATAGG GCAACCWCAAASAGACACCA GTTTTAGAGCTAGAAATAGC-3'
       <-------T7----------------><--target_sequence--><-------Primer------>
oligo 5 5'-GAAATTAATACGACTCACTATAGG GAGGTTCTTTGABTCCTTTG GTTTTAGAGCTAGAAATAGC-3'
        <-------T7----------------><--target_sequence--><-------Primer------>
oligo 6 5’-GAAATTAATACGACTCACTATAGG AAGGTGAACGTGGATGAAGT GTTTTAGAGCTAGAAATAGC-3'
        <-------T7----------------><--target_sequence--><-------Primer------>
oligo 7 5’-GAAATTAATACGACTCACTATAGG GCTCCTGGGCAACGTGC GTTTTAGAGCTAGAAATAGC-3'
        <-------T7----------------><--target_sequence--><-------Primer------>
oligo 8 5’-GAAATTAATACGACTCACTATAGG CAGAARGTGGTGGCTGGTG GTTTTAGAGCTAGAAATAGC-3'
```

B) Target sequences and oligo sequences for hemoglobin depletion

HBA targets	HBB targets
(1) AAGGSCCACGGCAAGAAGG	(1) CACTGTGACAAGCTGCACG
Human- AAGGGCCACGGCAAGAAGG	Human- CACTGTGACAAGCTGCACG
Polar- AAGGCCCACGGCAAGAAGG	Polar- CACTGTGACAAGCTGCACG
(2) CACTGCCTGCTGGTGACCC	(2) GAAGTTGGTGGTGAGGCCCT
Human- CACTGCCTGCTGGTGACCC	Human- GAAGTTGGTGGTGAGGCCCT
Polar- CACTGCCTGCTGGTGACCC	Polar- GAAGTTGGTGGTGAGGCCCT
(3) GGTYAAGGSCCACGGCAAGA	(3) CAGGCTGCCTATCAGAARG
Human- GGTTAAGGGCCACGGCAAGA	Human- CAGGCTGCCTATCAGAAAG
Polar- GGTCAAGGCCCACGGCAAGA	Polar- CAGGCTGCCTATCAGAAGG
(4) ACCTCCAAATACCGTTAAGC	(4) GCAACCWCAAASAGACACCA
Human- ACCTCCAAATACCGTTAAGC	Human- GCAACCTCAAACAGACACCA
Polar- ACCTCCAAATACCGTTAAGC	Polar- GCAACCACAAAGAGACACCA
(5) GCCGACAAGASCAACGTCA	(5) GAGGTTCTTTGABTCCTTTG
Human- GCCGACAAGACCAACGTCA	Human- GAGGTTCTTTGAGTCCTTTG
Polar- GCCGACAAGAGCAACGTCA	Polar- GAGGTTCTTTGACTCCTTTG
(6) GGGAAGTAGGTCTTGGTGGTG (r)	(6) AAGGTGAACGTGGATGAAGT
Human- GGGAAGTAGGTCTTGGTGGTG	Human- AAGGTGAACGTGGATGAAGT
Polar- GGGAAGTAGGTCTTGGTGGTG	Polar- AAGGTGAACGTGGATGAAGT
(7) TCCTRAGCCACTGCCTGC	(7) GCTCCTGGGCAACGTGC
Human- TCCTAAGCCACTGCCTGC	Human- GCTCCTGGGCAACGTGC
Polar- TCCTGAGCCACTGCCTGC	Polar- GCTCCTGGGCAACGTGC
(8) CAGGTCGCTCAGRGCGGACA (r)	(8) CAGAARGTGGTGGCTGGTG
Human- CAGGTCGCTCAGGGCGGACA (Lost PAM sequence)	Human- CAGAAAGTGGTGGCTGGTG
Polar- CAGGTCGCTCAGAGCGGACA	Polar- CAGAAGGTGGTGGCTGGTG

Figure S4. sgRNA design and construction.

Oligonucleotides designed for hemoglobin depletion from full-length cDNA. Oligos were chosen to deplete Hemoglobin mRNA transcripts from Human and Polar Bear whole blood. A) To generate sgRNAs a template free PCR was performed to anneal the tracrRNA oligo to an oligo containing the target sequence to generate a full-length oligo. The full-length oligos were converted into sgRNA templates using in-vitro transcription. B) Target oligos used for generating sgRNAs. Degenerate bases are highlighted in grey. (r) indicates reverse orientation

Sample Name	Sequencer	Run Type	Library Prep	Read Number	Median Accuracy
PB3_depleted	Illumina HiSeqX	2X151	Smart-seq2	22322746	N/A
PB19_depleted	Illumina HiSeqX	2X151	Smart-seq2	19418607	N/A
PB21_depleted	Illumina HiSeqX	2X151	Smart-seq2	22467660	N/A
PB3_undepleted	Illumina HiSeqX	2X151	Smart-seq2	58088942	N/A
PB19_undepleted	Illumina HiSeqX	2X151	Smart-seq2	105936701	N/A
PB21_undepleted	Illumina HiSeqX	2X151	Smart-seq2	63096050	N/A
PB19_undepleted	ONT MinION	9.4.1/LSK109	R2C2	5313	93%
PB3_depleted_R1	ONT MinION	9.4.1/LSK109	R2C2	390526	93%
PB3_depleted_R2	ONT MinION	9.4.1/LSK109	R2C2	1691780	94%
PB19_depleted_R1	ONT MinION	9.4.1/LSK109	R2C2	59097	93%
PB19_depleted_R2	ONT MinION	9.4.1/LSK109	R2C2	866087	97.5%
PB21_depleted	ONT MinION	9.4.1/LSK109	R2C2	830952	92%

Table S1: High-throughput sequencing runs and characteristics
For R2C2/ONT MinION runs, fully processed R2C2 read numbers and median accuracies are given. Some R2C2/ONT MinION runs were multiplexed, sometimes with samples unrelated to this study. Samples PB19_depleted_R2 and PB3_depleted_R2 represent the current output of the R2C2/ONT MinION combination

RT

>Oligo-dT-smartseq2
/5Me-isodC/AAGCAGTGGTATCAACGCAGAGTACTTTTTTTTTTTTTTTTTTTTTTTTTTTTTTVN
>TSO_Smartseq2
AAGCAGTGGTATCAACGCAGAGTACATrGrGrG

Primers for amplifying cDNA

>ISPCR
AAGCAGTGGTATCAACGCAGAGT

Tn5 Oligos (Smart-seq2 library prep)

>Tn5ME-R
[phos]CTGTCTCTTATACACATCT
>Tn5ME-A
TCGTCGGCAGCGTCAGATGTGTATAAGAGACAG
>Tn5ME-B
GTCTCGTGGGCTCGGAGATGTGTATAAGAGACAG

Primers for amplifying Tn5 Product

>Nextera_Primer_A
AATGATACGGCGACCACCGAGATCTACAC [8bp i5 index] TCGTCGGCAGCGTCAGATG
>Nextera_Primer_B
CAAGCAGAAGACGGCATACGAGAT [8bp i7 index] GTCTCGTGGGCTCGGAGATGTGTAT

R2C2 Splint_Oligos

>UMI_Splint_1_Forward_ISPCR (Matches ISPCR Primer)
ACTCTGCGTTGATACCACTGCTTTGAGGCTGATGAGTTCCATANNNNNTATATNNNNNATCACTACTTAGTTTTTGATAGCTTC
AAGCCAGAGTTGTCTTTTTCTCTTTGCTGGCAGTAAAAG
>UMI_Splint_1_Reverse_ISPCR (Matches ISPCR Primer)
ACTCTGCGTTGATACCACTGCTTAAAGGGATATTTTCGATCGCNNNNNATATANNNNNTTAGTGCATTTGATCCTTTTACTCCTC
CTAAAGAACAACCTGACCCAGCAAAAGGTACACAATACTTTTACTGCCAGCAAAGAG
>UMI_Splint_2_Forward_ISPCR (Matches ISPCR Primer)
ACTCTGCGTTGATACCACTGCTTTGCCGGTTGGGTATCAATAANNNNNTATATNNNNNATTGCCTTTATTCTATCTACTTAGTTT
TGGCGATGTAGTCTACCTATCCTGATGCTGAATAAAGGC
>UMI_Splint_2_Reverse_ISPCR (Matches ISPCR Primer)
ACTCTGCGTTGATACCACTGCTTAATTAGGTTCTAGGATCACGNNNNNATATANNNNNCTGCCATCGAAAATTTTTCACCCGTAA
CAAGAACTTACAACTCTCTGACGCCTATATCATGAAGGCCTTTATTCAGCATCAGGA

Table S2 Oligos used in the manuscript

All oligos are shown 5'->3' and were ordered from Integrated DNA Technologies (IDT). Lower case 'r' indicates RNA bases. Spaces in sequence
